# Permeability Transition Pore-Mediated Mitochondrial Superoxide Flashes Regulate Cortical Neural Progenitor Differentiation

**DOI:** 10.1371/journal.pone.0076721

**Published:** 2013-10-08

**Authors:** Yan Hou, Mark P. Mattson, Aiwu Cheng

**Affiliations:** 1 Laboratory of Neurosciences, National Institute on Aging Intramural Research Program, Baltimore, Maryland, United States of America; 2 Department of Neuroscience, Johns Hopkins University School of Medicine, Baltimore, Maryland, United States of America; University of South Florida, United States of America

## Abstract

In the process of neurogenesis, neural progenitor cells (NPCs) cease dividing and differentiate into postmitotic neurons that grow dendrites and an axon, become excitable, and establish synapses with other neurons. Mitochondrial biogenesis and aerobic metabolism provide energy substrates required to support the differentiation, growth and synaptic activity of neurons. Mitochondria may also serve signaling functions and, in this regard, it was recently reported that mitochondria can generate rapid bursts of superoxide (superoxide flashes), the frequency of which changes in response to environmental conditions and signals including oxygen levels and Ca^2+^ fluxes. Here we show that the frequency of mitochondrial superoxide flashes increases as embryonic cerebral cortical neurons differentiate from NPCs, and provide evidence that the superoxide flashes serve a signaling function that is critical for the differentiation process. The superoxide flashes are mediated by mitochondrial permeability transition pore (mPTP) opening, and pharmacological inhibition of the mPTP suppresses neuronal differentiation. Moreover, superoxide flashes and neuronal differentiation are inhibited by scavenging of mitochondrial superoxide. Conversely, manipulations that increase superoxide flash frequency accelerate neuronal differentiation. Our findings reveal a regulatory role for mitochondrial superoxide flashes, mediated by mPTP opening, in neuronal differentiation.

## Introduction

Mitochondria function as ‘cellular power plants’, generating adenosine triphosphate (ATP) that is required for cell survival and function. However, mitochondria have many other functions in addition to the production of ATP, including roles in signal transduction and the regulation of cellular calcium homeostasis [1]. Mitochondrial dysfunction is involved in pathogenesis of multiple neurological abnormalities including neurodegenerative disorders [2], hypoxic brain injury [3] and psychiatric disorders [4]. During the process of differentiation of neurons from neural progenitor cells (NPCs) mitochondrial biogenesis occurs to supply mitochondria for the growing dendrites and axon [5,6]. Because neurons are excitable cells and experience repeated bouts of membrane depolarization and Na^+^ and Ca^2+^ influx, they have a much higher aerobic metabolic rate than NPCs [7]. Because mitochondrial respiration generates superoxide anion radical, cells with greater amounts of active mitochondria, such as neurons and myocytes, produce more superoxide than non-excitable cells [1,8].

While excessive and uncontrolled production of superoxide and other reactive oxygen species (ROS) can result in cell injury and death [1], lower nontoxic levels play important roles in regulating multiple physiological cellular processes [9]. Oxygen availability regulates embryonic development via oxygen sensing pathways and intracellular redox state changes. Relatively low oxygen tension and low endogenous ROS levels maintain the viability and self-renewal capacity of stem cells [10]. Mitochondrial superoxide, generated during electron transport chain (ETC) activity, is the major source of intracellular ROS. We previously reported that excitable cells (cardiac myocytes and neurons) exhibit intermittent bursts of superoxide production (superoxide flashes), the frequency of which is modulated by oxygen tension [8]. In contrast, NPCs exhibit low levels of superoxide flashes, and when the superoxide flash frequency is increased the proliferation of the NPCs is reduced [11]. The mechanism by which mitochondrial superoxide flashes negatively regulate NPC proliferation involves inhibition of extracellular signal regulated kinases (ERKs) [11]. Collectively, the available data suggest that increased aerobic activity is associated with increased bursts of mitochondrial superoxide generation which, in turn, inhibits NPC proliferation. Because cessation of cell division is a prerequisite for neuronal differentiation, we determined whether superoxide flashes control the process of differentiation of NPCs into neurons.

In contrast to basal levels of superoxide production, mitochondrial superoxide flashes are triggered by opening of mitochondrial membrane permeability transition pores (mPTP) [8]. The molecular composition of the proteins comprising the mPTP has not been established, but increasing evidence points to the F(0)-F(1) ATP synthase (the last complex in the ETC) as being a major component of the mPTP [12]. A protein that is associated with mPTP is cyclophilin D, which is a molecular target of cycosporin A, a drug that inhibits mPTP opening [13]. Sustained mPTP opening mediates apoptosis, a form of programmed cell death in which cytochrome c is released from mitochondria and cell death effector proteins called caspases are activated [14]. In contrast, a transient “flickering” mode of mPTP opening can occur, and may play roles in ROS-mediated signaling and adaptive cellular stress responses [15,16]. The occurrence of superoxide flashes requires transient mPTP opening and ETC activity, suggesting a functional coupling of mPTP and the ETC [8]. In the present study we provide evidence that mPTP-mediated superoxide flashes promote the differentiation of neurons from NPCs.

## Materials and Methods

### Primary neural progenitor cell cultures and treatments

NPCs isolated from embryonic C57 mouse cerebral cortex were cultured as floating neurospheres as described previously [17]. Briefly, the telencephalon from embryonic day 14.5 mice was dissected in sterile Hanks’ balanced saline solution (HBSS). The collected cortical tissue was incubated in 0.05% trypsin-EDTA in HBSS for 15 min at 37°C and then transferred to culture medium which consisted of Dulbecco’s modified Eagle’s medium (DMEM)/F12 (1:1) supplemented with B-27 (InVitrogen, Carlsbad, CA) and 40 ng/ml fibroblast growth factor 2 (FGF2; Becton Dickson, Bedford, MA) and 40 ng/ml epidermal growth factor (EGF; InVitrogen, Carlsbad, CA). The cells were dissociated by trituration and then seeded at a density of 2 x10^5^ cells/ml in uncoated culture flasks. The formed neurospheres (2 days in culture) were dissociated and plated at 2.5 x 10^4^ cells/cm^2^ of culture surface on polyethylenimine (PEI) coated coverslips where the NPCs attached to the surface and continued to proliferate to form a monolayer NPC culture. Two days after plating the NPCs, the culture medium was replaced with medium lacking FGF2 and EGF (a condition which induces differentiation of the NPCs into neurons) and experimental treatments were added to the differentiation medium. Treatments included: 100 nM cyclosporine A (CsA; Sigma, St. Louis, MO); 1 µM atractyloside (ATR; Sigma, St. Louis, MO), 1 µM Mito-Tempol (Sigma, St. Louis, MO); and 1µM paraquat (PQ; Sigma, St. Louis, MO). All the agents were prepared as 1000x stocks in DMSO. Treatments were administered by direct dilution into the culture medium and an equivalent volume of vehicle was added to control cultures.

### Immunocytochemistry and imaging

Monolayer NPCs were fixed in a solution of 4% paraformaldehyde in PBS for 20 minutes. Cells were permeabilized and preincubated with blocking solution (0.3% Triton X-100 and 10% normal goat serum in PBS) for 30 min, and then incubated overnight at 4°C with antibodies against β-III tubulin (Tuj1; mouse, 1:250; Sigma, St. Louis, MO), Sox2 (1:200, Chemicon, Temecula, CA) and glial fibrillary acidic protein (GFAP; mouse, 1:250; Sigma, St. Louis, MO) antibodies. Then the cells were incubated with anti-mouse secondary antibodies (FITC-conjugated donkey IgG, 1:500, InVitrogen, Carlsbad, CA) in blocking solution for 2 h at room temperature. The cells were counterstained with 0.02% propidium iodide (PI) and 1% RNAse in PBS for 10 min; they were then washed with PBS and mounted on microscope slides in an anti-fade medium (Vector Laboratories, Burlingame, CA). To measure the percentage of each cell type, 300-500 cells in 4 random fields were counted, and the percentage of cells that were immunoreactive with each antibody was calculated. Images were acquired using a Zeiss LSM 510 confocal microscope with a 40X objective.

### Measurements of mitochondrial mass and function

Mitochondrial mass was measured in cells loaded with MitoTracker green (InVitrogen, Carlsbad, CA) using methods described previously [6]. Briefly, NPCs growing in 96-well plates were incubated with 50 nM MitoTracker green at 37°C for 30 min. Fluorescence intensity was measured using plate reader by excitation at 492 nm and emission at 515 nm and was normalized to protein concentration. For mitochondrial membrane potential (ΔΨ_m_), cells were incubated with 100 nM TMRE (InVitrogen, Carlsbad, CA) at 37°C for 20 min. Images were acquired with a Zeiss LSM 510 confocal microscope using a 40X objective lens with excitation at 579 nm and emission at 599 nm. Fluorescence intensity was measured using a plate reader and was normalized to MitoTracker green intensity [18]. Mitochondrial superoxide levels were measured with Mito-SOXRed (InVitrogen, Carlsbad, CA); cells were incubated with 1 µM Mito-SOXRed in HBSS containing 2 mM CaCl_2_, 1 mM MgCl_2_ and 10 mM glucose at 37°C for 10 min. Fluorescence intensity was measured using plate reader at 510 nm and emission at 580 nm and was normalized by protein concentration. Cellular ATP levels were measured using a kit according to the manufacturer’s protocol (Sigma, St. Louis, MO), and was normalized to protein concentration.

### Confocal imaging of mitochondrial superoxide flash generation

NPCs were transfected with 2 µg pcDNA3.1 plasmid containing mt-cpYFP coding sequence as described previously [11]. Confocal imaging used a Zeiss LSM 510 confocal microscope with a 63X, 1.3NA oil immersion objective and a sampling rate of 1.5 s / frame. Dual excitation imaging of mt-cpYFP was achieved by alternating excitation at 405 and 488 nm and emission at 505 nm. For each random field of live cells, a series of 100 images was acquired during a 150 s period. Scanning of each image took 1.57s. Imaging experiments were performed at room temperature (25±1°C). Digital image were analyzed using Zeiss LSM 510 software (Research Systems).

### Immunoblot analysis

Proteins in lysates of cultured cells were separated by SDS–PAGE electrophoresis (7.5–10% polyacrylamide gradient gel) and transferred electrophoretically to a nitrocellulose membrane. The membrane was blocked with 4% non-fat milk in a Tris–HCl based buffer containing 0.2% Tween 20 (pH 7.5), and then incubated in the presence of primary antibody overnight at 40°C followed by 1 h in the presence of a 1:5000 dilution of secondary antibody conjugated to horseradish peroxidase (Jackson Immunoresearch Laboratory, West Grove, PA). The membrane was further processed using an Enhanced Chemiluminescence Western blot detection kit (Amersham Bioscience, Piscataway, NJ). The primary antibodies (all from Sigma, St. Louis, MO) included those against cytochrome c (mouse, 1:1000), MnSOD (mouse, 1:1000), cyclophilin D (mouse, 1:1000) and actin (mouse, 1:1000).

### Data analysis

All data are presented as mean ± SD. Comparisons between control and treatment groups were performed using one way or two way ANOVA, with Bonferroni post-hoc test for pairwise comparisons. P < 0.05 was considered statistically significant.

## Results

### Mitochondrial superoxide flash frequency increases as neurons differentiate from NPCs

**Figure 1:**
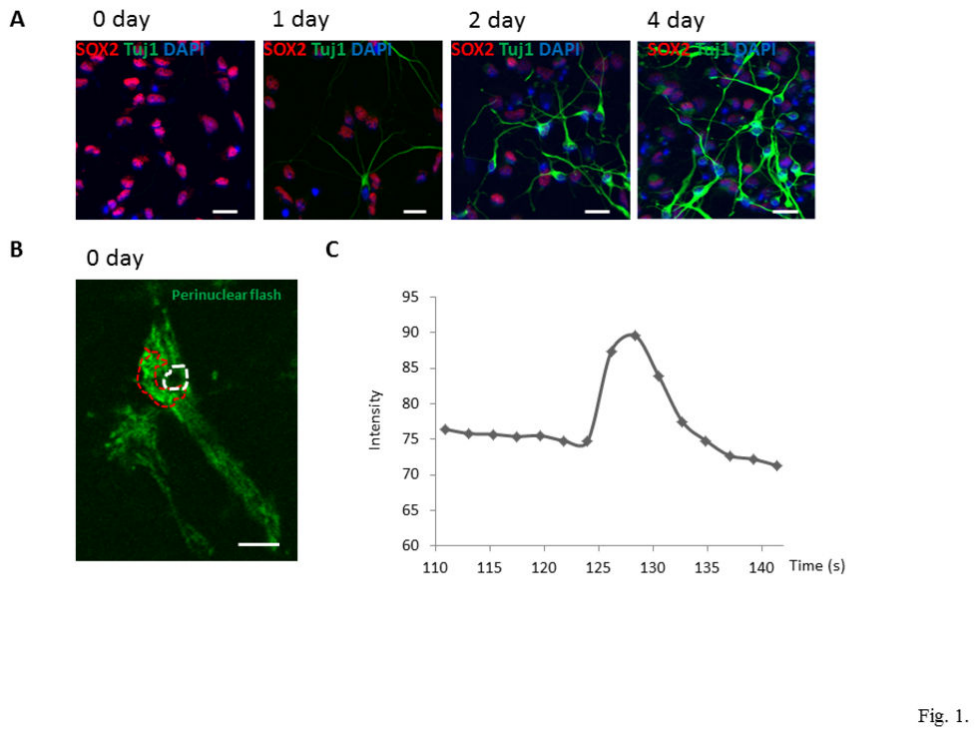
Characterization of mitochondrial SO flashes in NPCs. (A) Cell differentiation was induced by growth factor removal. NPCs and neurons were identified by antibodies against Sox2 (red) and Tuj1 (green), respectively, at different days of differentiation. The nuclei of NPCs were counterstained with DAPI (blue). Bar = 20 µm. (B-C) Characteristics of typical mitochondrial SO flashes in NPCs (day 0). The area circumscribed with the red dashed line is a perinuclear region where SO flashes occurred, and the area circumscribed with the white dashed line is the nucleus. Bar = 5µm (B). Change of mt-cpYFP fluorescence intensity during time course of a typical SO flash (C).

Primary NPCs were isolated from mouse cerebral cortex and cultured in the presence of FGF2 and EGF for 2 days. More than 98% of these cells expressed SOX2, nuclear protein known to be expressed in NPCs, but not in neurons or astrocytes. Differentiation of the NPCs was induced by depriving the cells of FGF2 and EGF. Similarly to what was reported previously [19], more than 90% of the cells expressed SOX2 on day1 of differentiation, about 75% of the cells expressed SOX2 on day 2, and less than 30% of the cells expressed SOX2 on day 4. There was a significant decrease in percentage of SOX2-positive cells as well as increase in percentage of Tuj1-positive cells during differentiation [One way ANOVA: SOX2: F(3, 12) =607.53, p<0.001; Tuj1: F(2, 9) =242.90, p<0.001] (Figure 1A, Figure S1). Tuj-1-positive neurons were identified as early as day1 of differentiation, whereas GFAP-positive astrocytes were not observed until day 2 (Figure S1). NPCs exhibited a round or elongated morphology with one or two flattened processes, whereas Tuj-1-positive neurons exhibited multiple long, thin neurites. NPCs transfected to express the Mt-cpYFP superoxide biosensor exhibited a weak basal superoxide signal that revealed mitochondria that were relatively short and arranged in a reticulum-like manner in a perinuclear location, and as elongated individual mitochondria in the cytoplasmic extensions (Figure 1B).

As reported previously [11], mitochondrial superoxide flashes were infrequent in NPCs, with approximately 11% of the NPCs exhibiting one or more SO flashes during a 150 s imaging period. The superoxide flashes were characterized by a transient increase in mt-cpYFP fluorescence. Figure 1B shows a typical SO flash occurring in perinuclear mitochondria of NPCs (day 0). Figure 1C depicts kinetic changes of mt-cpYFP fluorescence intensity during this typical perinuclear flash, which arose abruptly within 2-3 s and returned to baseline level within 10 s (Figure 1C). On day 0 (before differentiation), the intensity of the SO flashes peaked in 2.8 ± 0.6 s (mean ± SD of 60 flashes, Tp, time to peak fluorescence intensity) and dissipated with a half-time of 4.08 ± 1.2 s (mean ± SD of 60 flashes; T50, 50% decay time after the peak). The average fractional peak increase of mt-cpYFP fluorescence (ΔF/F0) during flashes of NPCs was 0.26 ± 0.12 (mean ± SD of 60 flashes).

**Figure 2:**
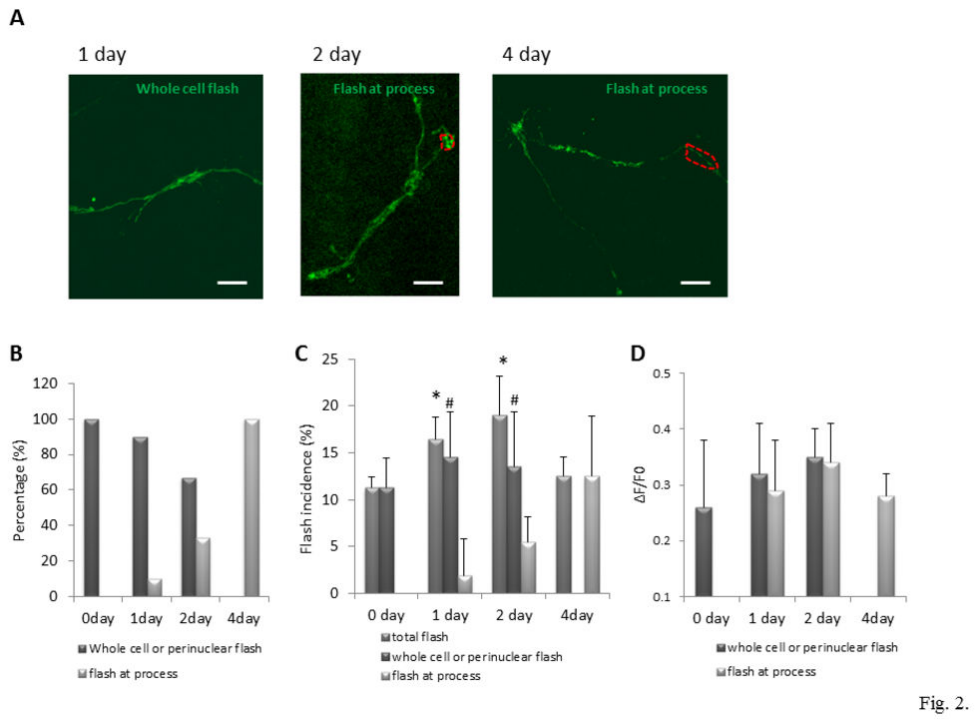
Characterization of mitochondrial SO flashes in NPCs during differentiation. (A) Distribution of mitochondrial SO flashes in NPCs at different days of differentiation. Left, middle and right panels show typical flashes (high intensity fluorescence) in mitochondria of cells on differentiation days 1, 2 and 4. See Movies S1 and S2 for a dynamic view of the superoxide flashes. Bar = 5 µm. (B) Flash pattern changes during NPC differentiation indicated by percentages of flashes occurring in mitochondria throughout the whole cell, in perinuclear regions or in processes/neurites, among all flashes detected. n=60-100 flashes (from four independent cultures) analyzed on each individual day of differentiation. (C) Flash incidence in NPCs during cell differentiation. Flash incidence refers to percentage of cells exhibiting mitochondrial superoxide flashes among all cells scanned during the imaging period (n=4 independent experiments and approximately 150-210 cells scanned per experiment on each individual day of differentiation). Values are expressed as mean ± SD. *p<0.05 vs. total flash incidence of NPCs at day 0; #p<0.05 vs. incidence of flashes in whole cell or perinuclear region at day 0. (D) Flash amplitude (ΔF/F0) in NPCs during differentiation. Flash amplitude was calculated as the ratio ΔF /F0. ΔF indicates difference between peak fluorescence intensity and basal fluorescence intensity. F0 refers to basal fluorescence intensity. Values are expressed as mean ± SD. 60-100 SO flashes (from 4 independent experiments) analyzed on each individual day of differentiation.

The cells (day1) exhibited mitochondrial superoxide flashes that occurred simultaneously throughout the whole cell or in a perinuclear region (left panel of Figure 2A; Movie S1), suggesting the existence of a mechanism for synchronizing superoxide flashes among closely associated mitochondria. During the process of neuronal differentiation, as newly generated neurons elaborated neurites, the percentage of cells exhibiting whole-cell superoxide flashes decreased and flashes occurred in group of mitochondria within neurites (middle and right panel of Figure 2A; Movie S2). All superoxide flashes (100%) detected in NPCs (day 0) occurred throughout whole cell or in perinuclear mitochondria. At day 1 of differentiation, 90% of the flashes occurred in the whole cell or in a perinuclear location, and 10% flashes occurred in mitochondria located in processes. At day 2 of differentiation, about 67% of the flashes occurred within perinuclear regions or whole cells; while 33% of the flashes occurred in mitochondria in the processes. By differentiation day 4, all of flashes (100%) occurred in processes (60-100 flashes analyzed for each day of differentiation). Flash incidence in NPCs at day 0 was 11.2 ± 1.2% (mean ± SD of 600-850 cells analyzed). Total flash incidences (flashes occurring in whole cell, perinuclear region and processes) in cells on differentiation days 1 and 2 were significantly increased by 46% and 68%, respectively, compared to NPCs on day 0 (Figure 2C). The incidence of superoxide flashes in whole cell and perinuclear region were also significantly increased by 29% and 20%, respectively, compared to flash incidence of NPCs on day 0. This result indicates that superoxide flashes occurring in mitochondria in processes also contribute to the robustly increased flash incidence on days 1 and 2 of differentiation. On day 4 of differentiation all flashes occurred in mitochondria located in processes (Figure 2C) [One way ANOVA: F(3, 12) =5.17, p<0.05]. The amplitude of perinuclear or whole cell superoxide flashes was greater on differentiation days 1 and 2 (23% and 35% increase, respectively) compared to that on day 0 (NPCs of day 0: ΔF/F0 =0.26 ± 0.12; Figure 2D). However, those differences in amplitude of perinuclear or whole cell superoxide flashes during differentiation were not statistically significant [One way ANOVA: F(2, 9) = 1.18, p=0.35]. On differentiation day 4 the amplitude of individual flashes had receded to the same level as that in NPCs (day 0).

### Mitochondrial biogenesis during neuronal differentiation is associated with enhanced mitochondrial membrane polarization and Mn-SOD levels

**Figure 3:**
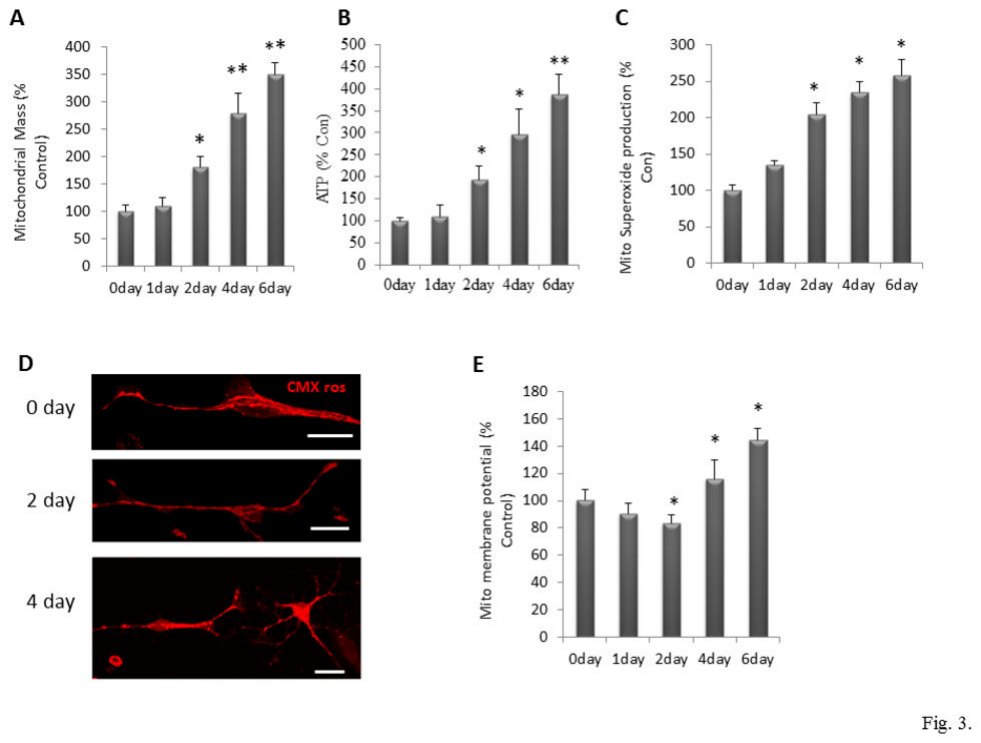
Mitochondrial mass and function changes in NPCs during differentiation. (A, B and C) Mitochondrial mass, measured by Mitotracker green loading (A), mitochondrial ATP content (B) and mitochondrial superoxide production, evaluated by MitoSOXred loading (C) are normalized by protein concentration in differentiated cells. (D) Mitochondrial membrane potential (∆Ψm) was imaged by mitoCMXros loading in NPCs on differentiation days 0, 2 and 4. Bar = 20 µm. (E) Mitochondrial membrane potential (∆Ψm) was determined by the ratio of MitoCMXros to Mitotracker green intensity. Values are expressed as a percentage of the mean of NPCs at day 0 (n = 4 separate experiments performed on NPCs cultured from 4 pregnant mice) *p<0.05, **p<0.001 compared to the values of NPCs at day 0.

A progressive increase in mitochondrial biogenesis occurs during the differentiation and growth of newly generated neurons [6]. We evaluated mitochondrial mass by measuring MitoTracker Green fluorescence levels (normalized to the total cellular protein level) in NPCs (day 0) and in cells on differentiation days 1, 2, 4 and 6. There was a highly significant progressive increase in mitochondrial mass from culture days 1 through 6 (Figure 3A) [One way ANOVA: F(4, 15) = 136.76, p<0.001]. The increase in mitochondrial mass was paralleled by progressive increases of cellular ATP levels (Figure 3B) [One way ANOVA: F(4, 15) = 40.38, p<0.001] and mitochondrial superoxide levels (Figure 3C) [One way ANOVA: F(4, 15) = 82.14, p<0.001]. The time courses and relative magnitudes of increases in mitochondrial biogenesis and ATP levels were similar, suggesting that newly generated mitochondria were fully functional. To determine whether mitochondria in newly generated neurons exhibited any differences from those in NPCs, we measured mitochondrial membrane potential (ΔΨ_m_) using the fluorescent probe TMRE. During the first 2 days of differentiation there was a significant 20% decrease of the ΔΨ_m_, followed by significant 18% and 42% increases in ΔΨ_m_ on differentiation days 4 and 6, respectively (Figure 3D, E) [One way ANOVA: F(4, 15) =18.04, p<0.001].

**Figure 4:**
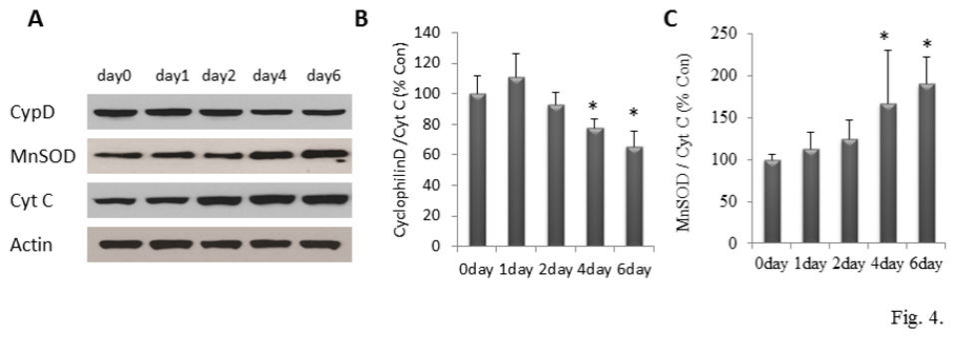
Expression of mitochondrial proteins in NPCs during differentiation. (A, B and C) Immunoblot analysis was performed by using antibodies that selectively recognize cytochrome c, MnSOD and cyclophylin D. Blots were reprobed with antibodies against Actin. Panel A shows representative blots; panels B and C show results of densitometric analysis of cyclophylin D and MnSOD, which were normalized to the corresponding mitochondrial cytochrome c level on each differentiation day. Values are expressed as a percentage of the mean of NPCs at day 0 (n = 3-4 separate experiments performed on cells cultured from 3-4 pregnant mice); *p<0.05 compared to the values of NPCs at day 0.

Immunoblot analysis revealed that levels of the mitochondrial protein cytochrome c increased during differentiation (Figure 4A, B), consistent with increased mitochondrial mass during differentiation. Levels of cyclophilin D (CyP-D), an essential component of mPTP that regulates ΔΨ_m_, significantly decreased at day 4 and day 6 during differentiation (Figure 4A, B) [One way ANOVA: F(4, 15) =9.45, p<0.001], which suggests and elevation of ∆Ψm during late differentiation. Levels of manganese superoxide dismutase (MnSOD), a mitochondrial enzyme that converts superoxide to hydrogen peroxide, increased progressively during the 6 day period of cell differentiation, with the level nearly doubling by day 6 (Figure 4A, C) [One way ANOVA: F(4, 15) =5.33, p<0.05].

### Evidence that mPTP activity and associated superoxide production promote neuronal differentiation

**Figure 5:**
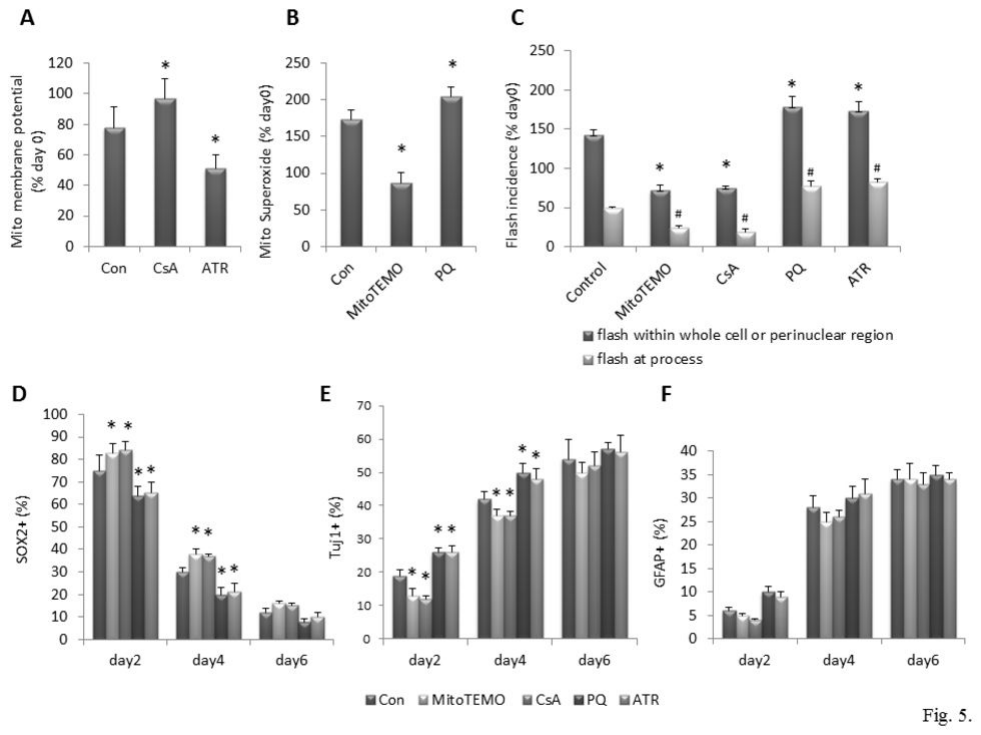
Mitochondrial superoxide flashes promote NPC differentiation. (A) Mitochondrial membrane potential in differentiated cells at day 2. Cells were treated with 100 nM CsA, 1µM ATR or vehicle at the time of growth factor removal. Values are expressed as a percentage of the mean of the control condition in NPCs (day 0). *p<0.05 compared to the control value in cells (day 2). (B) Mitochondrial superoxide in differentiated cells at day 2. Cells were treated with 1µM Mito-Tempol (MitoTEMO), 1µM PQ or vehicle at the time of growth factor removal. Values are expressed as a percentage of the mean of the control condition in NPCs (day 0), *p<0.05 compared to the control value in cells (day 2). (C) Superoxide flash incidence in differentiated cells at day2. Cells were treated with CsA, ATR, Mito-Tempol and PQ as indicated in A and B. Values are expressed as a percentage of the mean of the control condition in NPCs (day 0); Perinuclear or whole cell flash: *p<0.05 compared to the control value in cells (day 2); Flash at process: #p<0.05 compared to the control value in cells (day 2). (D, E and F) Percentage of NPCs, neurons and astrocytes at different days of differentiation. NPCs, neurons and astrocytes were identified by antibodies against Sox2, Tuj1 and GFAP at different days after differentiation. Total number of cells was counted by counterstaining nuclei with DAPI. n = 4 separate experiments performed on NPCs cultured from 4 pregnant mice. *p<0.05 compared to the control value of each individual day.

Emerging evidence that mitochondrial superoxide flashes serve a signaling function in the regulation of processes such as cell proliferation [11], stress responses [20] and apoptosis [21]. We therefore performed experiments to determine whether conditions that modify mitochondrial superoxide flashes can affect the differentiation of neurons from NPCs. Our previous study indicated that mitochondrial SO flash generation requires a functional coupling of transient mPTP opening with a burst of mitochondrial SO production [11]. Reagents inhibit or enhance mPTP opening and mitochondrial SO production were employed to manipulate SO flashes at day 2 of differentiation, a time point with the most robust SO flash activities (Figure 2C, 2D). Concentrations of the pharmacological agents that were non-toxic were selected based on data from previous studies [11]. As expected, treatment of cells with 100 nM cyclosporine A (CsA), an inhibitor of the mPTP, increased mitochondrial membrane potential (Figure 5A) and reduced superoxide flash incidence (Figure 5C). Conversely, treatment of cells with 1 µM atractyloside (ATR), an agent that promotes opening of mPTPs, reduced mitochondrial membrane potential (Figure 5A) and increased superoxide flash incidence (Figure 5C). In addition, we found that mitochondrial superoxide generation and mitochondrial superoxide flash incidence were reduced in cells treated with the mitochondrial superoxide scavenger mitoTEMPO (1 µM). In contrast, mitochondrial superoxide production and mitochondrial superoxide flash incidence were enhanced in cells exposed to 1 µM paraquat (Figure 5B, 5C). To determine whether mitochondrial superoxide flashes modify the process of neuronal differentiation, NPCs were treated on differentiation day 0 with vehicle (control), mitoTEMPO, CsA, paraquat or ATR. On differentiation days 2, 4 and 6 cells in each group were then immunostained with antibodies against markers of NPCs (SOX2), neurons (Tuj1) and astrocytes (GFAP). The percentage of cells that were SOX2-positive on differentiation days 2 and 4 was significantly greater in cultures treated with MitoTEMPO or CsA compared to control cultures (Figure 5D). The percentage of cells that were SOX2-positive on differentiation days 2 and 4 was significantly lower in cultures treated with paraquat or ATR compared to control cultures (Figure 5D) [Two way ANOVA: mitoTEMPO : F(1, 18) =12.36, p<0.05; CsA: F(1, 18) =12.98, p<0.05; paraquat : F(1, 18) =21.73, p<0.001; ATR: F(1, 18) =15.76, p<0.001, respectively]. The percentage of cells that were Tuj1-positive on differentiation days 2 and 4 was significantly lower in cultures treated with MitoTEMPO or CsA compared to control cultures (Figure 5E). The percentage of cells that were SOX2-positive on differentiation days 2 and 4 was significantly greater in cultures treated with paraquat or ATR compared to control cultures (Figure 5E). By differentiation day 6, the percentage of cells that were SOX2 positive had decreased to 8-12% and there were no significant differences among the experimental treatment groups, although there were trends towards increased SOX2+ cells in cultures treated with MitoTEMPO and CsA, and decreased SOX2+ cells in those treated with paraquat and ATR (Figure 5D) [Two way ANOVA: mitoTEMPO : F(1, 18) =9.73, p<0.05; CsA: F(1, 18) =8.78, p=0.008; paraquat : F(1, 18) =19.07, p<0.001; ATR: F(1, 18) =8.82, p<0.05, respectively]. By differentiation day 6 over 50% of the cells were Tuj-1 positive in all cultures with no significant differences among the groups (Figure 5E). GFAP+ cells comprised approximately 35% of the cells in all treatment groups on differentiation day 6 (Figure 5F).

## Discussion

Our findings reveal that during the process of differentiation of NPCs into neurons, mPTP-mediated mitochondrial superoxide flash generation increases. By exposing NPCs to agents that either inhibit or enhance mitochondrial superoxide flash generation, we provide evidence mPTP opening and superoxide flash generation promotes the neuronal differentiation process. Previous studies have shown that excitable cells, including neurons, cardiac myocytes and skeletal muscle cells exhibit high rates of mitochondrial superoxide flash generation [8,22]. On the other hand, we have found that mitochondria in non-excitable mitotic cells, including NPCs [11] and astrocytes (A. Cheng, unpublished data), generate relatively few superoxide flashes. Generation of mitochondrial superoxide flashes may explain the previously reported effects of cellular redox status on the fate of embryonic stem cells; pro-oxidative conditions enhance differentiation into neurons and cardiac myocytes, and reducing conditions promote self-renewal of the stem cells [23]. Similarly, exposure of NPCs to mild oxidizing conditions promotes neuronal differentiation, whereas reducing conditions decreases neurogenesis [24]. We found that mitochondrial superoxide flashes negatively regulate the proliferation of cerebral cortical NPCs by a mechanism involving inhibition of ERK MAP kinases [11]. The latter finding is consistent with prior evidence that ERK promotes NPC proliferation [25,26], and that a protein called Spred1, an endogenous inhibitor of the Ras – ERK pathway suppresses the proliferation of neural stem cells in the ventricular zone [27].

We found that the differentiation of NPCs into neurons is inhibited by blocking of the mPTP with cyclosporin A. Similar to our findings, CsA inhibits the differentiation of hematopoietic progenitor cells [28]. Others have reported that CsA increases the number of NPCs in the subventricular zone, possibly by increasing the survival of NPCs rather than inhibiting their differentiation [29]. On the other hand, CsA can enhance the differentiation of natural killer cells from hematopoietic progenitors [30], enhances differentiation of cardiac cells from induced pluripotent stem cells [31], and inhibits the proliferation of vascular progenitor cells [32]. The concentration of CsA and the duration of exposure of cells to CsA varied considerably among previous studies, which may influence whether CsA promotes or inhibits the proliferation and differentiation of stem cells. Because the existence of mPTP-mediated mitochondrial superoxide flashes was only discovered recently [8], the possibility that effects of CsA on stem cell fate involved superoxide had not been considered. Our finding that treatments that reduce mitochondrial superoxide levels inhibit the differentiation of NPCs into neurons, suggest that transient openings of mPTPs may regulate the fate of NPCs by a superoxide flash-mediated mechanism. Our findings that treatment of cells with MitoTEMPO which reduces mitochondrial superoxide levels inhibits the differentiation of NPCs, whereas treatment with paraquat which enhances mitochondrial superoxide levels promotes differentiation of NPCs, further supports a key role for superoxide flashes in regulating differentiation.

Consistent with previous studies of monkey stromal stem cells and human mesenchymal stem cells [33,34] we found that, in self-renewing NPCs, mitochondria are located in a perinuclear cluster. As neurons differentiate from the NPCs the mitochondria increase in number and move into the neurites. Cellular ATP levels increase progressively as neurons grow their axons and dendrites suggesting that mitochondrial respiratory function is increased to sustain the higher energy demand of excitable neurons. Previous studies have shown that during neurogenesis there is a switch in cellular energy production from anaerobic glycolysis in NPCs to aerobic mitochondrial oxidative phosphorylation in differentiated neurons [35]. The reverse metabolic shift occurs when induced pluripotent stem cells are derived from somatic cells [36]. When taken together with the latter findings, our data suggest that increased bursts of mitochondrial superoxide production are associated with the switch in cellular energy metabolism. We found that the ΔΨ_m_ was relatively low in NPCs and during the first 2 days of NPC differentiation and then was significantly elevated on differentiation days 4 and 6. As neuronal differentiation proceeded, levels of cyclophilin D decrease, whereas levels of MnSOD increased. Altogether, our findings therefore suggest that mitochondrial superoxide flash generation increases during neuronal differentiation, and that those flashes are actively involved in promoting the cell differentiation process.

## Supporting Information

Figure S1 **Percentage of SOX2, Tuj1 and GFAP positively stained cells at different days of differentiation (days 0 to 4).** NPCs, neurons and astrocytes were identified by antibodies against SOX2, Tuj1 and GFAP, respectively. Total numbers of cells were determined by counting DAPI-stained nuclei. n = 4 separate experiments performed on NPCs cultured from 4 pregnant mice. *p<0.05 compared to the percentage of cells stained with SOX2 at day 0. #p<0.05 compared to the percentage of cells stained with Tuj1 at day 1.
(PDF)

Movie S1 **Example of superoxide flashes occurring in groups of mitochondria in newly-generated neurons.**
(AVI)

Movie S2 **Example of superoxide flashes in individual mitochondria located in neurites.**
(AVI)
