# Supplementary figures and images for: Permeability Transition Pore-Mediated Mitochondrial Superoxide Flashes Regulate Cortical Neural Progenitor Differentiation
Source: PLoS One. 2013 Oct 8;8(10):e76721. doi: 10.1371/journal.pone.0076721 (PMC3792897; doi:10.1371/journal.pone.0076721)

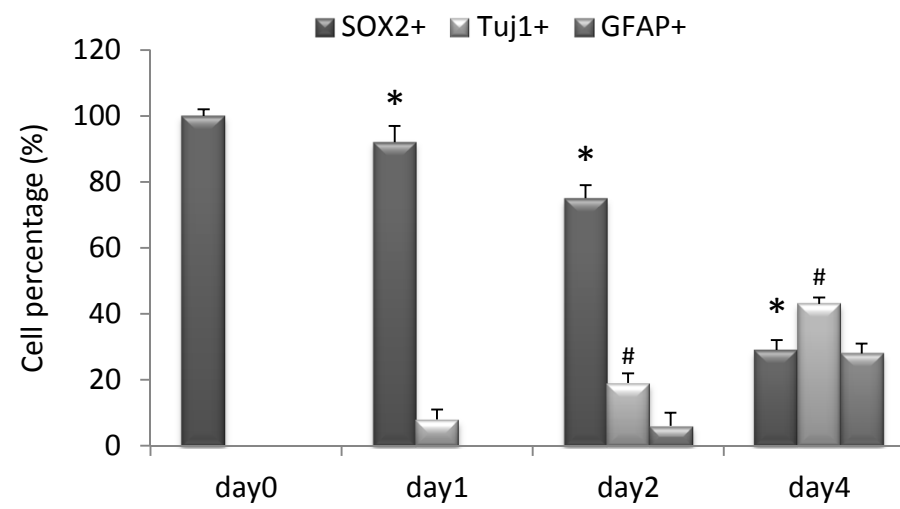

Fig. S1.

Supplement: Figure S1 — Percentage of SOX2, Tuj1 and GFAP positively stained cells at different days of differentiation (days 0 to 4). NPCs, neurons and astrocytes were identified by antibodies against SOX2, Tuj1 and GFAP, respectively. Total numbers of cells were determined by counting DAPI-stained nuclei. n = 4 separate experiments performed on NPCs cultured from 4 pregnant mice. *p<0.05 compared to the percentage of cells stained with SOX2 at day 0. #p<0.05 compared to the percentage of cells stained with Tuj1 at day 1. (PDF) [file pone.0076721.s001.pdf]
